# Role of workplace bullying and workplace incivility for employee performance: Mediated-moderated mechanism

**DOI:** 10.1371/journal.pone.0291877

**Published:** 2024-01-30

**Authors:** Shahid Mehmood, Maham Rasool, Masood Ahmed, Hossam Haddad, Nidal Mahmoud Al-Ramahi

**Affiliations:** 1 Department of Management Sciences, Faculty of Management Sciences and IT, Mohi-Ud-Din Islamic University Nerian Sharif, AJ&K, Islamabad, Pakistan; 2 MS Management Sciences, Riphah International University, Islamabad, Pakistan; 3 Department of Public Administration, Faculty of Management Sciences, University of Kotli, AJK, Kotli, Pakistan; 4 Business Faculty, Zarqa University, Zarqa, Jordan; 5 Business Faculty, Accounting Department, Zarqa University, Zarqa, Jordan; Czestochowa University of Technology: Politechnika Czestochowska, POLAND

## Abstract

Workplace events play a significant role in shaping the performance of employees and organizations. Negative events, in particular, require careful attention due to their severe impact on employee wellbeing and performance. Workplace bullying and incivility are two negative events that can cause significant harm to employees and contribute to poor performance. This study examines the effects of workplace bullying and incivility on employee performance in the presence of perceived psychological wellbeing (PWB), with the moderating role of perceived organizational support (POS) considered for both independent variables and employee performance (EP). The study focuses on female nurses working in the healthcare sector of Azad Jammu and Kashmir. The results of the study indicate that workplace bullying and incivility have adverse effects on employee performance and that PWB mediates these relationships. Moreover, perceived organizational support moderates the relationship between both predictors and the employee performance criterion. In conclusion, the findings of this study highlight the importance of creating a positive and supportive work environment to mitigate the negative effects of workplace bullying and incivility on employee performance.

## Introduction

A working environment with good behaviors of coworkers and supervisors is a source of satisfaction and good performance [1]. But, the involvement of comportments like using abusive language, name-calling, blustering, bullying, and mild to high levels of deviant behaviors can cause dissatisfaction among workers and can compromise their performance [2]. Workplace bullying is becoming a serious concern for all organizations because it is considered a stressor that negatively affects the psychological and physical well-being of the target object [3]. Workplace bullying includes any act like harassing or offending someone by targeting their psychological, physical, or workplace well-being [4]. The term ‘bullying” is a sum up of many terms like violence, physical or mental abuse, provocation, and workplace hostility or harassment [5]. This topic remains under study for many years and still has significant importance especially in the healthcare sector. Within the Health care sector, para-medical staff, especially nurses, play a significant role in providing high-quality care to patients [1]. Many incidents of workplace bullying are reported in the healthcare sector, about 20% rise in workplace bullying is observed in USA hospitals during last few years [6]. A recent study conducted in the hospital of Karachi have shown prevalence of workplace bullying in health care sector and results have shown that 53% males and 38% of females are victims of bullying [7]. These figures shows that the situation is becoming alarming therefore there is an urgent need to address this issue. On the basis of previous researches and reviews it is assumed that healthcare sector have more ratio of workplace bullying cases as compare to non-healthcare sectors [8].

Sometimes workplace bullying is accompanied by workplace incivility. Workplace incivility includes all those deviant behavior with unclear or ambiguous intentions to harm others [9]. Workplace incivility is noticed everywhere in every sector. Therefore, it is becoming more important to take this factor under study. A strong connection between workplace bullying and workplace incivility was found by [10]. Workers are many times let down by their coworkers due to their incivility and bullying which risks the overall performance of the victim [11]. Such sort of behaviors like mistreatment, ill mannerism, nasty comments, and disrespectful behaviors causes dissatisfaction among workers. Dissatisfaction from the workplace causes low performance. Researchers have found that incivility brings a rise in intention to quit a job, absenteeism, and a decrease in organizational commitment among nurses [12]. Sometimes nurses feel themselves unsafe and helpless at hospital or health care units due to the ineffective government policies against such uncivil activities. Researchers have directed that due to bullying and incivility the professional and psychological well-being of nurses is on the stack [5]. Bullying causes depression, anxiety and insomnia into the target which can have devastating effect on their psychological wellbeing. From researches it is also pointed out that nurses getting targeted by incivility or bullying show low performance, scattered confidence and shattered personalities [13]. Therefore, it is becoming a need to address this issue to mitigate the consequences of bullying and incivility in healthcare or non-health care sector.

Numerous researchers have worked on bullying in the workplace and its consequences by taking nursing staff under study [14–16] but the focus of those researches were only the consequences and prevalence of workplace bullying despite of abundant of researchers still this issues seems un addressed because reported cases of bullying have increased in numbers. Recently [8] have conducted a study on the prevalence, antecedents, and consequences of bullying on nurses. Farley et al. 2023; Ren and Kim 2023, have studied the effect of bullying on well-being of employees but leaders are failed to choose a more comprehensive approach to eliminate these issues at workplace which means there is still a dearth of studies on this topic. Although, workplace bullying have so many devastating effects on workplace but most important of all is performance of workers. Performance of worker is main source of efficient and effective working of the organization [17]. So, in the light of previously conducted studies current study has formulated a new model to understand mediating moderating mechanism. Current study have aimed to observe the interplay of mediators and moderators by testing indirect effects of workplace bullying and incivility on the job performance.

This study will contribute to literature in several ways firstly, a significant approach is used by having psychological wellbeing as a mediator between work place bullying, incivility and employee’s performance and by exploring moderating role of perceived organizational support. Secondly, the current study will deepen our understanding of workplace bullying and incivility at workplace by taking support from affective event theory (AET). Affective event theory proposes that employees reactions are based upon the ongoing event happens at the workplace [18]. Routine work hassles and emotions of the employees can be trigger their performance. Workplace happening can have strong positive and negative effects on the employees. Therefore, we are using this theory in this study we explored that how nurse’s performance is effected when uncivil act or bullying by supervisors and coworkers happens at the workplace. This study will have a practical contribution by helping policy makers in the development of bullying and incivility preventing interventions and strategies which might include building positive working environment by inducing healthy competitions among worker and creating a goal oriented environment.

## Hypothesis development

From literature it is directed that workplace bullying and incivility has significant negative impacts on nurses. Deviant behaviors at the workplace is having detrimental consequences on the physical and psychological wellbeing of nurses [8]. Incidents of bullying causes distress , depression and demotivation among nurses which lowers down their self-esteem and leads them towards showing low performance [13]. Abundant of researches were conducted on health care sector every researcher has indicated an inverse relationship between the employees work performance and deviancy like bullying and incivility at workplace [14, 19, 20]. It is also directed that it depends upon the culture and environment of the organization, that how these events are reinforced or controlled [21].

Victims of bullying seek support from the HR department to cop up the situation but if they get no support it lowers down their moral and their commitment towards their organization shrinks [22], on the other hand if employees feel that hospital are admiring their work and their contribution and the management listens to their grievances then nurses feel themselves more valuable [23]. On the basis of literature we have developed hypothesis which are given below.

### Workplace bullying and employee performance

Workplace bullying is defined any sort of unwanted and aggressive, repetitive actions done to hostile or humiliate someone and enhancing threat, low, depression and distress in the target. It is termed as an unethical and negative behavior [24]. According to [25] bullying is a conscious repetitive conduct happens to harm others. Bullying may occurs when power and status difference exists between the target and bully, it may happen from the side of supervisor or peer [26]. From literature it is retrieved that workplace bullying can happen in horizontal and vertical orders. Horizontal bullying happens from peers and coworkers, workers from the same rank form group and target others whereas in vertical bullying subordinates are targeted and supervisor are involved [27]. It is further categorized into two folds first one is, work base bullying which means when the worker is overburdened by putting tremendous work pressure And second one is harassment which includes spreading rumors, indirect name calling, using abusive language and double meaning jokes [28]. When bullying by coworkers and supervisors happens on regular basis the bullied person goes through less job satisfaction low performance.

Performance is defined as routine activities performed by a worker according to the given job description. An organizational culture supporting bullying brings low performance, it severely effects leadership and conducive working environment [28]. According to [29] employees performance has strong link with the culture and environment of organization. Workplace bullying is a strong predictor of employee low productivity because sometimes employee’s performance is measured in the form of level of productivity employees shows at workplace. And a negative relationship between the employee productivity and workplace bullying is found [27]. Any types of exploitations by coworkers and supervisors lower downs motivation level of the victim, creates a mental physical pressure on them which eventually effects an individual’s capabilities to perform [30]. According to [31] Long term bullying and negative conducts build a pressure on employees and leads to low efficiency and performance. While discussing performance we can see that workplace bullying crumbled the motivation level of employees and become a hurdle in the way of high performance [32]. Based on the above discussion we have hypothesized that

***H***_***1***_ **: *Workplace bullying has negative impact on workplace bullying***

### Workplace incivility and employee performance

Workplace incivility is defined as low intensity deviant behaviors with ambiguous intent to harm others [33]. Incivility has become a topic of discussion as it is noticed in every organization and in every sector [34]. Workplace incivility occurs when workers disrespects others in any way and violate the rules of the organization [35]. Incivility could happen in any form, verbal or nonverbal or by any one, coworker or supervisors [36]. When showing incivility at the workplace employees avoid interaction with each other and they don’t share the important information with their colleagues which effects individual and well as organizational performance [37]. Abundant of researches were conducted on workplace incivility and all the negative outcomes of incivility were found by the researchers like, low productivity, low job commitment, low organizational loyalty, self-blaming, badly effected psychological and physical wellbeing [25, 38–41]. A person targeted by the others incivility feels annoyed by the others and start taking more leaves and therefore, his individual performance gets effected badly [42]. Which means that the targets become involve in work avoiding behaviors. Incivility creates an unfriendly working environment leading workers toward depression and psychological distress which reduces their productivity and low performance [37].

From the discussion we have hypothesized that

***H***_***2***_ **: *Workplace incivility has negative effect on employee performance***

### Mediating role of psychological well being between workplace bullying and employee performance

Psychological wellbeing is defined as effective psychological functioning and how a person feels about his life is known as psychological wellbeing [43]. Pleasant state of mind a person have towards his overall life is also considered as psychological wellbeing [44]. Employee psychological wellbeing is considered as a predictor of various job outcomes therefore, it is essential for organizations to focus on psychological wellbeing of employees so that maximum performance could be gained from workers [45]. Previous researchers have found many mediating role of psychological wellbeing.

According to [46] employees facing negativity at workplace have lower level of self-esteem and psychological wellbeing. Whereas job performance, bullying and psychological wellbeing have close relations. Psychological wellbeing is measured by the job satisfaction and work related depressions which has been posited as a mediator between stressor performance relationship [17]. Anxiety, depression and dissatisfaction are consequences of bullying and which have obvious effects on employees’ performance [31]. But psychological wellbeing is considered a source of happiness for employees, when psychological well- being of employees is present then motivation level of employees also increases which is vital for organizational performance [47]. Researchers have indicated that psychological wellbeing can work as a mediator between bullying and performance. Studies have shown strong negative link between workplace bullying and job satisfaction and correspondingly solid researchers have evidence that satisfaction and work related depressions are predictor of employee performance [48, 49].Whereas presence of psychological wellbeing can mitigate the effects of bullying on employees. On the basis of strong grounds build up by literature psychological wellbeing can mediate the relationship between bullying and performance. Psychological wellbeing can convert a depressive workplace to happy working environment by enhancing job satisfaction among workers therefore, we have hypothesized following relationship of psychological wellbeing

***H***_***3*:**_ ***Psychological wellbeing mediates the relationship between workplace bullying and employee performance***

### Mediating role of psychological well being between workplace incivility and employee performance

Psychological wellbeing is considered as an important element of workplace. It is defined as continues professional and personal grooming of a person by all means [50]. Psychological wellbeing is measured on the basis of many parameters in the past, Physical and mental health, social and organizational supports and satisfaction with life [47, 51, 52]. Employees work related attitudes are effected by their psychological wellbeing [53]. When workplace is not peaceful and employees breach the norms of the workplace it is incivility which negatively effects the performance of the workers. Most of the time people quit their job when they came in contact with any type of incivility [54]. Workplace Incivility has both work related and non-work related consequences, it harms the employees expectations regarding supervisors and colleagues which effects their performance [55]. Any sort of toxicity by supervisors and coworkers costs wellbeing of employees [45]. Disturbed psychological wellbeing causes low performance, low decision making power and decreases the satisfaction level [56].

[57, 58] have found that psychological wellbeing is considered as important factor to predict employee performance because higher wellbeing increases individual and organizational performance. Psychological wellbeing is considered as a source of happiness because of which productivity and performance of employees increases [59]. From the above literature it is claimed that psychological wellbeing can play role of mediator because incivility at workplace effects psychological wellbeing of employees and effected wellbeing reduces the work performance.

So is has hypothesized that

***H***_***4***_ **: *Psychological wellbeing mediates the relationship between incivility and employee performance*.**

### Perceived organizational support as a moderator

Perceived organizational support is defined as assumptions of employees regarding organization that how the organization value their contributions and the extent to which it is concerned about their wellbeing [60]. Workplace bullying and incivility both have gain so much publicity in workplace literature due to their devastating consequences, that some organizations have designed policies against these deviant behaviors [61]. Abundant of researches have examined that does POS mitigates the effects of bullying on employees.

Researchers have found positive association between perceived organizational support and employee performance. [62] have conducted a study on POS and its effects on wellbeing and performance of employees the results have shown a positive association between POS and employee performance. Similar studies were conducted by [58, 63] and they have found that when employees feel support from organization it will increase their satisfaction about workplace and they will move in batter directions. Recently [64] have studies the link between POS and bullying and have found that on the presence of POS the effects of bullying and negative workplace behavior reduces due to high level of satisfaction. [65] have used POS as a moderator to lower down effects of bullying on employees.

From literature we have proposed that POS can serve as a moderator which can diminish the effects of uncivil and deviant behaviors on employee wellbeing and performance. Therefore, hypotheses for current research are:

***H***_***5***_: ***Perceived Organizational Support moderates the relationship in between workplace bullying and employee performance***.
***H***_***6***_: ***Perceived Organizational Support moderates the relationship in between workplace incivility and employee performance***.

**Fig 1:**
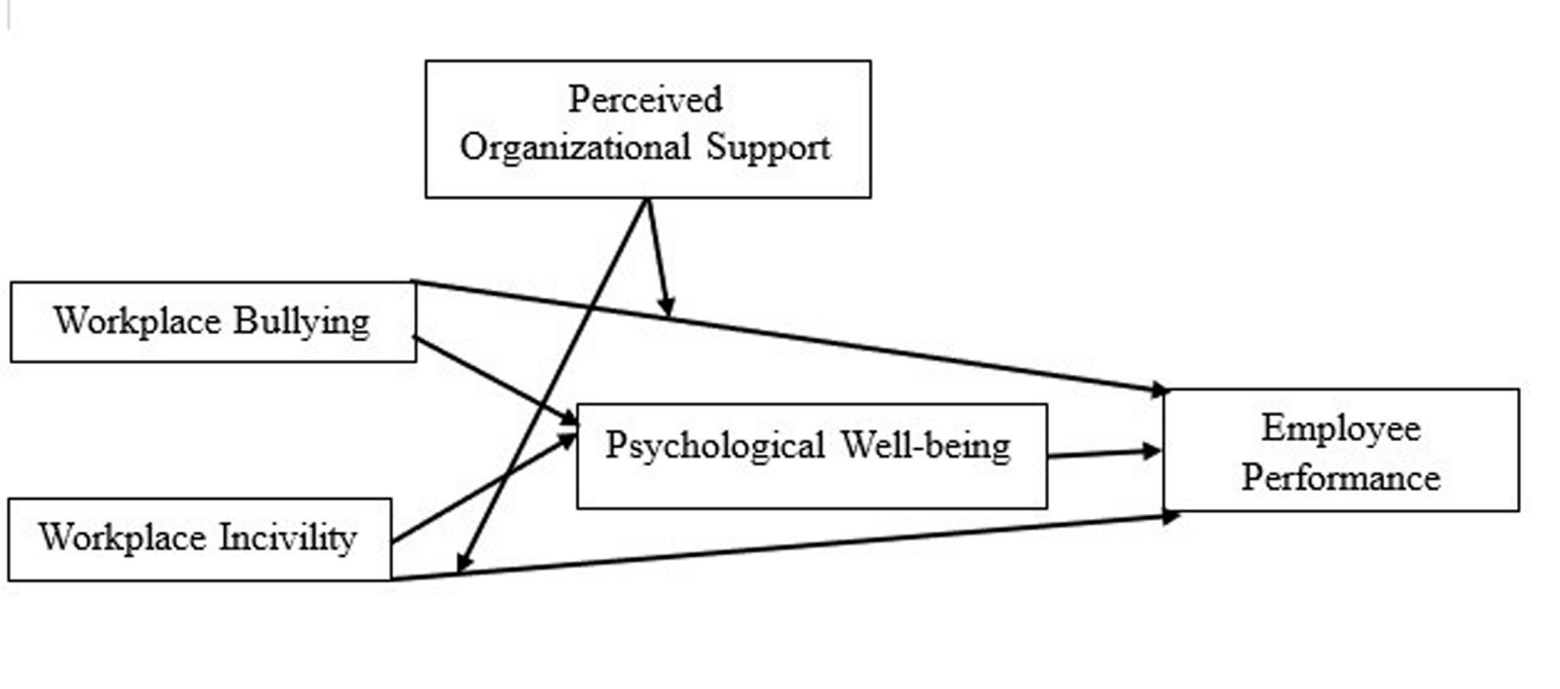
Research model.

See Fig 1: Research Model.

## Materials and methods

### Research design

Selection of appropriated methodology and research design is most crucial and important stage of the research process [66]. This makes possible to answer the research questions in efficient and effective manner [67]. Social sciences are widely dependent on quantitative research approach and help the researchers to analyze the data with the help of numbers [66]. As in current research self-administered questionnaires were used to collect the data, therefore quantitative techniques are deployed to analyze the data.

### Population and sample

Current research study targeted the health sector of AJ&K, Pakistan as population and all the female nurses and their immediate bosses are targeted to get response for the research study. In mathematical perspectives of research, researchers use random sampling for the research process [67], and in most of cases in research probability techniques of sampling are not supportive when we have to approach the total population targeted for any kind of research study. Therefore, in such kind of research studies we have to focus on non-probability sampling [68], this enables the researchers to have greater chances of selection from members of whole population. Nurses in healthcare institution is most busy worker and her duties are so much sensitive, as they are responsible for treatment and care of the patients, such situation pushed the researchers to select the sample on the basis of convenience. Nurses that were found available conveniently during the survey were selected as sample. Hence, total 1200 female nurses were selected as sample on the basis of convenient sampling technique.

### Measures

To collect the data questionnaire was designed by adopting the scales of variables involved in current research. Scale for workplace bullying is adopted that consist of 22-items and was developed by [69], this scale is also known as Negative Acts Questionnaire. To get the response the scale was anchored by point five likert scale that ranges from Never-1 to Daily-5. To collect the response for workplace incivility self-reporting scale developed by, [70] was used and is comprised of 9-items. In current research responses for mediator psychological well-being were gathered by using GHQ 12-items scales developed by [71]. Another variable perceived organizational support in current study is moderator and 36-items scale for data collection for this variable is used that has been developed by [72]. Scales for WB, WI, PW and POS were self-reporting while to collect data for performance of nurses working in health sector AJ&K, supervisor rated scale is adopted and was developed by [73, 74] it is comprised of 21 items.

### Data collection

To collect the data, we have distributed the self-administered questionnaires to the female nurses. *To ensure the ethical consideration*, *the written consent was obtained from the University of Kotli*, *AJK Ethics Committee to interact and gather data from the respondents for research purpose*. *Therefore*, *respondents (only volunteers) were informed and engaged for data collection through the written consent apprised by the University Ethical Committee*. *Hence*, *consent was informed through officially approved whilst annexed written statement with questionnaire*. Further, the considered study is not retrospective in nature, nor it gazed at archived samples. In first phase of data collection female nurses were invited to give their response for workplace bullying, workplace incivility, psychological well-being and perceived organizational support. After collecting these responses with the use of back to back coding on questionnaire, in phase-II supervisors or immediate bosses of female nursing staff were requested to give their response for the performance of female nurses. As the data were collected in two different phases so the longitudinal survey method was used in this research for data collection. Longitudinal survey method for data collection helps researchers to reduce the chances of common technical biasness [75]. In order to collect the data for current research 1200 Questionnaires were distributed to the sampled members and 912 members have provided their response. Out of 912 it had been found that 39 questionnaires were filled with less attention and few were incomplete as well and these 39 questionnaires were found inappropriate for data analysis. Hence, for data analysis 873 questionnaire were recorded in in data sheet. Response rate was found 73% in current research study.

## Results

### Data analysis

Research has used AMOS 21 to analyze the data and SEM was deployed to analyze the relationship among observed and latent variables of the current research study. As per [76] SEM is two phase process and first phase there is measurement and in other there is structural model. Measurement model is to be use for the confirmatory factor analysis (CFA) before SEM. According to [77] when the relationship among proposed relationships and selected patterns are stronger, the maximum model-fit can be obtained between model data. Reliability was measured to check the internal consistency of the scales used in current research and found reliable. To get the scales reliable the value of Cronbach Alpha must be greater than 0.70 in social sciences [78]. Pearson correlation was also measured among the variables and it should not be greater than 0.85 but greater than this value can be accepted if when the pertaining variables, relationships or model has any theoretical support [79]. After checking the correlation, we had tested the hypotheses of current research. Lastly, we have examined the mediating role and moderating role.

## Instrument’s validity

Average variance extract (AVE) is referred to the sum square of all of the factor loadings divided by the total number of items. The acceptable value of AVE i.e., the convergent validity, is >0.50 [79]. However as per findings of [80] values for AVE or convergent validity are also acceptable if they are < 0.50, but in this case the composite reliability must be greater than 0.60. Another way to measure the validity of construct is to check the discriminant validity of construct. The degree of construct can be examined with the help of discriminant validity. Discriminant validity (DV) refers to the difference occurs in one construct with respect to other. Further, DV can be calculated by taking square root the AVE or convergent validity and value of DV must be greater than AVE value of the dimension [79].

Therefore, as per results of current research, values of AVE and DV for scale of workplace bullying are found 0.50 and 0.63 respectively and are in acceptable range (Table 1). Another independent variable in current research is workplace incivility and AVE value for scale is 0.56 and value of discriminant validity is 0.68 (Table 2) and are above the benchmark values. Psychological wellbeing is the mediating variable in current research and was measured through GHQ-12 items and AVE value of scale was found 0.52 and DV value of this scale was found 0.62 (Table 3), hence this scale is found good enough to use. Perceived Organizational support is a moderator in this research study its scale was also found valid to use as its value for AVE is 0.55 and for DV is 0.61, both values are in acceptable range (Table 4). Finally, validity test was performed for the scale of dependent variable i.e., employee performance and convergent validity (AVE) is found 0.51 and the value for DV is 0.58 (Table 5).

**Table 1:** Validity of WB.

Name of Variable	Items	Factor Loading	Item Decision	AVE Score	CR Values	DV Values
**WB**				0.50	0.93	0.63
	WB1	.51	Included			
	WB2	.72	Included			
	WB3	.65	Included			
	WB4	.66	Included			
	WB5	.74	Included			
	WB6	.67	Included			
	WB7	.66	Included			
	WB8	.68	Included			
	WB9	.70	Included			
	WB12	.69	Included			
	WB13	.62	Included			
	WB14	.64	Included			
	WB15	.52	Included			
	WB16	.71	Included			
	WB17	.65	Included			
	WB19	.51	Included			
	WB20	.56	Included			
	WB21	.52	Included			
	WB22	.56	Included			

**Table 2:** CFA of WI.

Name of Variable	Items	Factor Loading	Item Decision	AVE Score	CR Values	DV Values
**WI**				0.56	0.88	0.68
	WI1	.73	Included			
	WI2	.70	Included			
	WI3	.76	Included			
	WI4	.71	Included			
	WI5	.67	Included			
	WI6	.64	Included			
	WI7	.50	Included			
	WI8	.62	Included			
	WI9	.71	Included			

**Table 3:** Validity of PW.

Name of Variable	Items	Factor Loading	Item Decision	AVE Score	CR Values	DV Values
**PW**				0.52	0.81	0.62
	PW1	.73	Included			
	PW2	.59	Included			
	PW3	.72	Included			
	PW4	.60	Included			
	PW5	.65	Included			
	PW7	.52	Included			
	PW9	.51	Included			

**Table 4:** Validity of POS.

Name of Variable	Items	Factor Loading	Item Decision	AVE Score	CR Values	DV Values
**POS**				0.55	0.92	0.61
	POS1	.66	Included			
	POS2	.57	Included			
	POS3	.71	Included			
	POS4	.69	Included			
	POS5	.56	Included			
	POS6	.61	Included			
	POS8	.60	Included			
	POS9	.51	Included			
	POS12	.71	Included			
	POS14	.52	Included			
	POS15	.53	Included			
	POS16	.62	Included			
	POS23	.56	Included			
	POS24	.59	Included			
	POS26	.54	Included			
	POS29	.57	Included			
	POS31	.59	Included			
	POS34	.56	Included			
	POS35	.65	Included			
	POS36	.55	Included			

**Table 5:** Validity of EP.

Name of Variable	Items	Factor Loading	Item Decision	AVE Score	CR Values	DV Values
**EP**				0.51	0.89	0.58
	EP1	.52	Included			
	EP3	.61	Included			
	EP5	.52	Included			
	EP6	.56	Included			
	EP7	.56	Included			
	EP8	.51	Included			
	EP10	.61	Included			
	EP11	.60	Included			
	EP12	.54	Included			
	EP13	.51	Included			
	EP14	.63	Included			
	EP15	.51	Included			
	EP16	.62	Included			
	EP17	.59	Included			
	EP18	.57	Included			
	EP19	.57	Included			

## Reliability analysis

Reliability analysis was performed to check the internal consistency of scales and **Table 6** is showing Cronbach alpha value for WB (0.926), for WI (0.881), for POS (0.915), for PW (0.788), and the value of Cronbach alpha for EP is found 0.881. As all the values are greater than 0.70, hence acceptable. All the scales adopted in current research were found reliable to conduct the research by using them.

**Table 6:** Reliability analysis.

Variable	Reliability	No of Items
WB	0.926	19
WI	0.881	9
POS	0.915	20
PW	0.788	10
EP	0.881	16

## Model fit summary

Considering the after exclusion values of Table 7 it is clear that values of each variable of goodness of fit index (GFI), incremental fit index (IFI), comparative fit index (CFI), normal fit index (NFI), and Tuker-Lewis fit index (TLI) in current research were found greater than 0.90 hence the values are acceptable. Table 2 is showing that values of RMSEA for all variables in current research are found in acceptable range as all values are less than 0.08. To determine the model fitness the acceptable criteria is: the fit indices must be greater than 0.90 [81–83], while the RMSEA should be less than 0.08 [84].

**Table 7:** Model fit summary.

	Results								
	WB*	WB**	WI	PW*	PW**	POS*	POS**	EP*	EP**
χ2 / d.f.	5.41	3.24	3.01	5.01	2.41	5.01	3.91	5.10	2.90
GFI	0.89	0.92	0.99	0.39	0.91	0.79	0.99	0.59	0.96
IFI	0.67	0.95	0.92	0.89	1.10	0.81	1.00	0.71	0.90
CFI	0.78	1.01	0.90	0.71	0.91	0.69	1.01	0.88	0.93
NFI	0.59	0.99	1.11	0.38	0.94	0.81	1.21	0.69	1.10
TLI	0.57	0.91	1.02	0.49	1.12	0.77	0.99	0.71	1.42
RMSEA	0.13	0.05	0.03	0.11	0.07	0.09	0.06	0.11	0.06

* Before Exclusion

** After Exclusion

## Correlation analysis

Correlation analysis was performed to check the relationships and direction of relationships among the pertaining variables of current research. Relationship of WB is found significant and positive with WI, significant and negative with PW and for EP the relationship is found also negative and significant. Association of WI is positive and significant with POS, as per results shown in Table 8 relationship of WI is found significant and negative with EP in current research. POS in current research has positive and significant relationship with PW and negative significant with EP. Lastly, as per correlation table, Table 3 PW has negative and significant relationship with EP.

**Table 8:** Correlation analysis.

	Age	Exp	Edu	WB	WI	POS	PW	EP
Age	1							
Exp	.518^**^	1						
Edu	.179^**^	.354^**^	1					
WB	-.065	-.055	.051	1				
WI	-.079^*^	-.099^**^	.010	.524^**^	1			
DTPM	-.017	-.076^*^	-.067^*^	-.107^**^	-.059			
DTPN	.033	.009	-.035	-.164^**^	-.144^**^			
DTPP	.052	.000	-.032	-.090^**^	-.113^**^			
POS	-.015	-.022	-.095^**^	.228^**^	.203^**^	1		
PW	.094^**^	.062	.068^*^	-.159^**^	-.170^**^	-.077^*^	1	
EP	-.008	.002	-.047	-.236^**^	-.200^**^	.168^**^	-.212^**^	1

**. Correlation is significant at the 0.01 level (2-tailed).

*. Correlation is significant at the 0.05 level (2-tailed).

## Direct relationships

### Workplace bullying to employee performance

**Fig 2:**
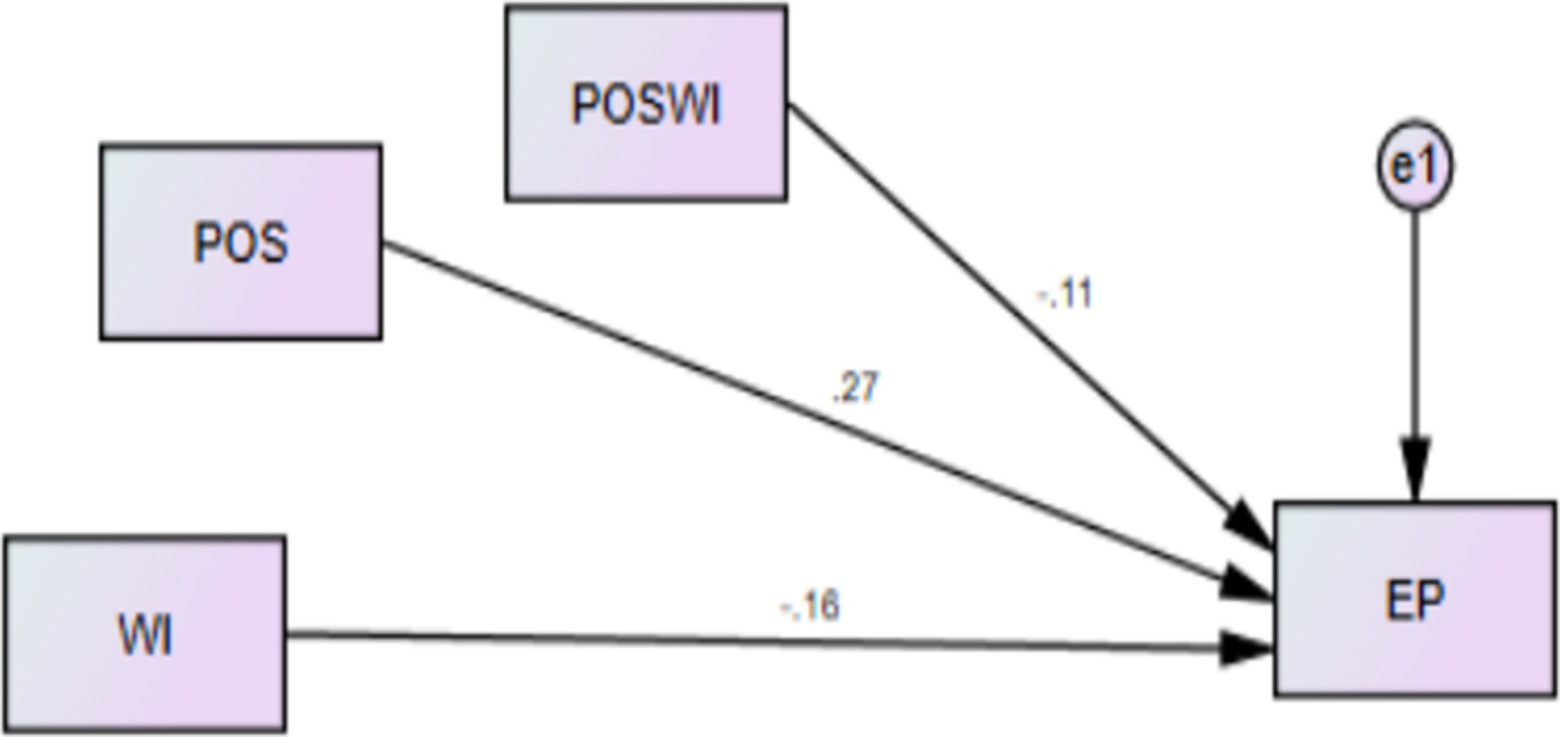
Direct relationships of WB-EP and WI-EP.

Table 9 and Fig 2 both are showing the results for direct relationship of WB with EP and WI with EP. Results revealed that WB has negative and significant effect on performance of female nurses working in health sector of Azad Jammu and Kashmir, Pakistan. Hence H1 of current research is supported by results and results of current research for H1 are in line with the studies of [75, 85, 86], they found that prevalence of bullying is higher in health sector as compared to other sectors and WB has negative consequences for productivity, performance and satisfaction of the employees or workforce.

**Table 9:** Direct relationships of WB-EP and WI-EP.

	Estimate	C.R.	P
WB-→EP	-.18	-5.539	.000
WI-→EP	-.11	-3.204	.001

### Workplace incivility to employee performance

Results in current study depicted that WI has negative and significant relationship for EP. This shows at in presence of workplace incivility in the health sector of AJ&K, Pakistan, female nurses are found less efficient and productive. Their performance is found low in the presence of WI. Thus, the H2 of the research study is supported by the results. Results for H2 of the study are consistent with the findings of past study conducted by [87]. Therefore prevalence of WI is not good symbol for organizations.

## Indirect relationship

**Fig 3:**
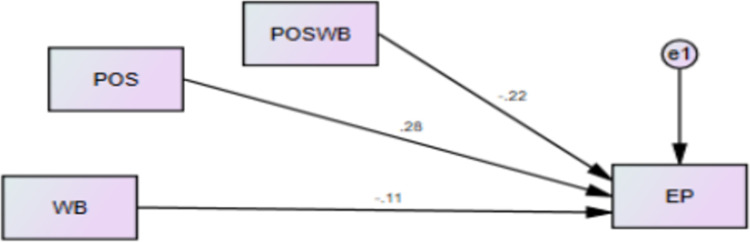
Indirect relationship.

Mediating role of PW between the relationship of WB and EP was tested, results revealed that psychological well-being mediates the relationship. When there is WB and WI in the organizations, employees are found more stressed, their mental health is found low, hence this cause low psychological among the nurses and finally their performance is not satisfactory in this situation. Role of PW in the presence of WB and WI for EP is decent contribution in literature by authors and the specific model was not tested in past studies with support of Affective Events Theory [18]. According to the statistical values shown in Fig 3 and Table 10 the H3 and H4 of current study are supported.

**Table 10:** Mediating role of PW.

Results of Indirect Effect	Estimate	LL 95% CI.	UL 95% CI
WB→PW→EP	0.0407	0.0188	0.0640
WI→PW→EP	0.0431	0.0221	0.0672

## Moderating role POS between WB and EP

**Fig 4:**
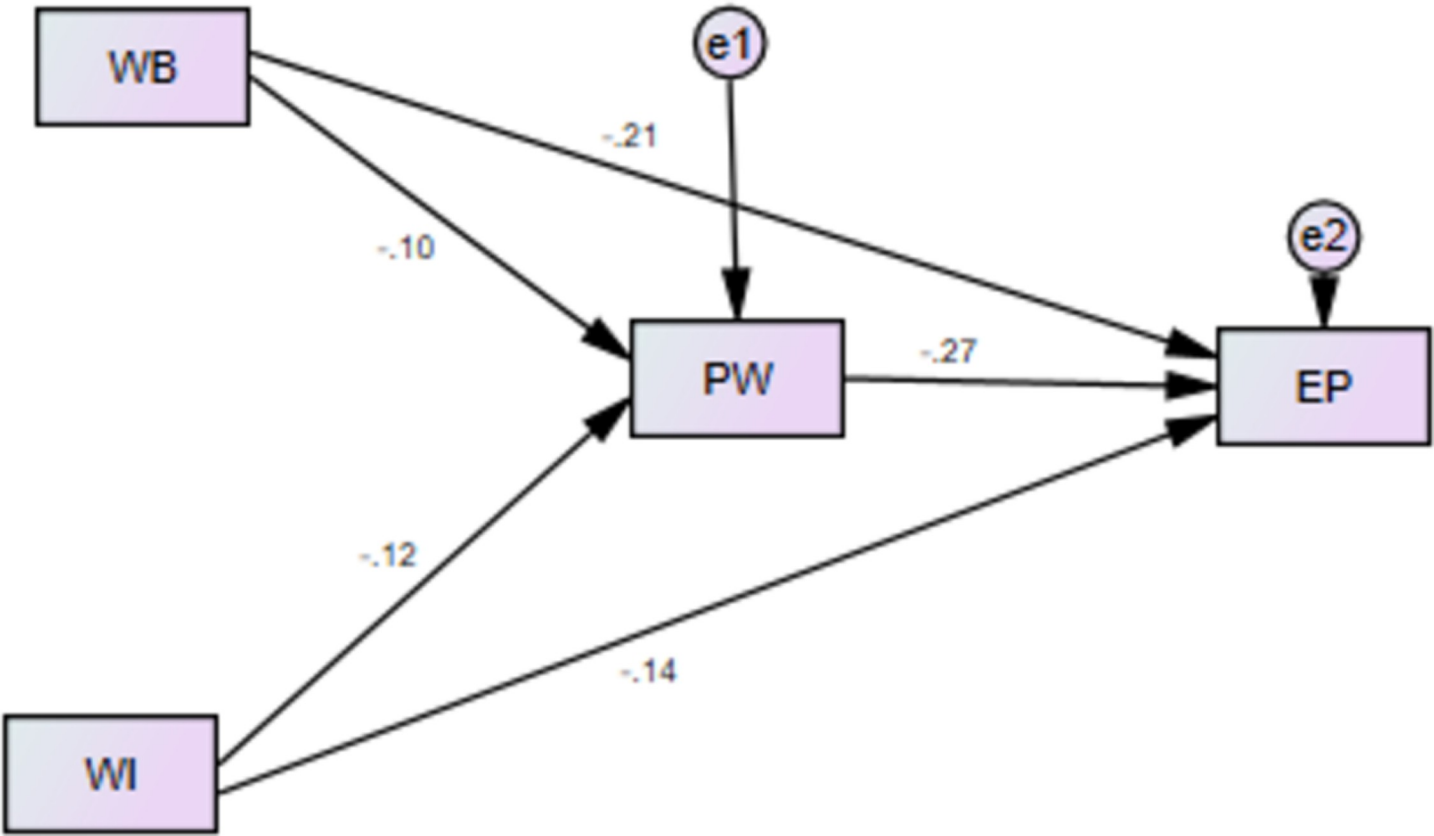
Moderating effect of POS.

Moderating role of POS is also tested for relationship of WB and EP. Although POS is used by different researchers as moderator in different context but not tested for the relationship of WB and EP. To test this relationship H5 was formulated in current research. As per results of current study, Fig 4 and Table 11 are showing that POS acts as moderator between WB and EP. In current research findings revealed that POS is not sufficient to minimize the effects of WB, therefore prevalence of WB has drastic outcomes even with the moderating role of POS. POS is supportive in many cases to help employees to reduce the effects of negative events but in the case of health sector of AJ&K, Pakistan POS is found less supportive to eradicate the bullying and its consequences for the performance of female nurses of frontline workers in healthcare units. Anyhow, study has proven that POS moderates the relationship of WB and EP, thus H5 of current study is supported by results. This contribution is another novelty in current research which has filled the theoretical and contextual gaps in literature.

**Table 11:** Moderating role POS between WB and EP.

	Estimate	C.R.	P
WB→EP	-.43	-14.709	.000
POS→EP	.09	3.079	.002
WB*POS→EP	.24	8.045	.000

## Moderating role POS between WI and EP

**Fig 5:**
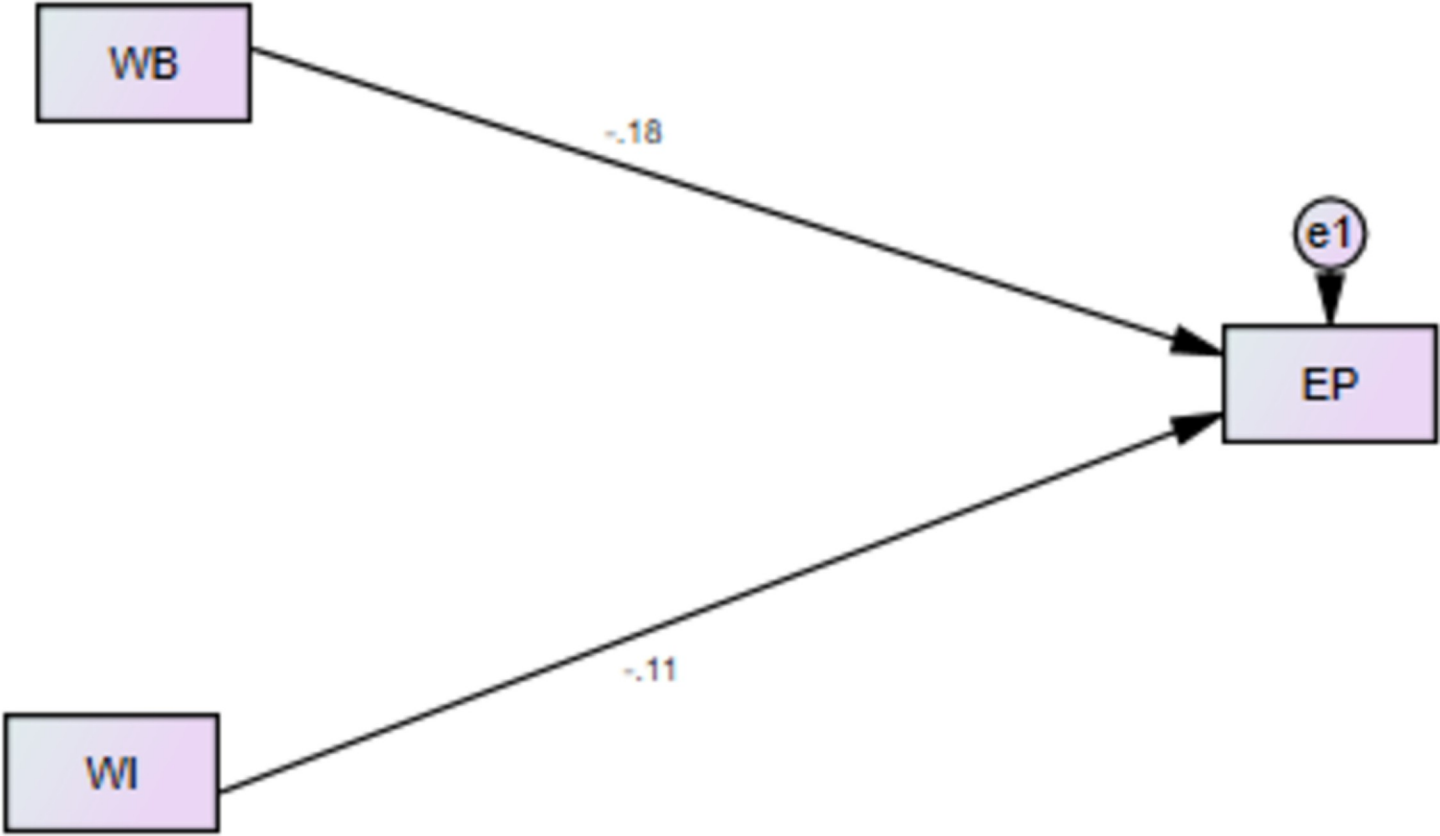
Moderating effect of POS.

To test the moderating role of POS between the relationship of WI and EP, H6 was formulated in current research. Results in Fig 5, and Table 12 have shown that alone POS has positive effect for employee performance but as moderator between the relationship of WI and EP, POS has not enough strength to eradicate the consequences of WI among female nursing staff in healthcare units of AJ&K, Pakistan. However, as per results it is right to report that POS has moderating effect for the relationship of WI and EP. Hence, this helped us to prove our H6 of the current research study. Support of H6 enabled us to contribute for literature contextually and theoretically which is third novelty of current research.

**Table 12:** Moderating role POS between WB and EP.

	Estimate	C.R.	P
WI→EP	-.16	-5.111	.000
POS→EP	.27	8.594	.000
WI*POS→EP	-.11	-3.472	.000

## Conclusion

The major focus of current research was to examine the effects of negative events at workplaces on the performance of employees. To address research questions and to meet the research objectives we have chosen female nursing staff and their supervisors working in health sector of AJ&K, Pakistan as population. The relationships of WB and WI with EP. To examine this H1 and H2 were formulated. It has been found in current research that bullying and incivility both has drastic results for performance of healthcare staff and specifically female nurses that are working in healthcare units situated in AJ&K, Pakistan. Thus, H1 and H2 of the research study is supported by results. Results regarding both hypotheses are consistent with the findings of [17, 88–91]. All the studies reported that negative events like bullying and incivility are not good for any organization regardless of its nature. As per this study especially healthcare organizations and specifically female nurses or front-line female workers suffer a lot due to the prevalence of negative events (WB and WI) at the healthcare units of AJ&K, Pakistan.

Secondly, mediating role of PW has also been tested and to test this role H3 and H4 was formulated. Results revealed that psychological wellbeing mediates the relationship of workplace bullying and employee performance. Similarly, mediating role of psychological wellbeing between workplace incivility and employee performance was tested and as per results PW mediated the relationship. Therefore, H3 and H4 of the research study got support from results. Mediating role of psychological wellbeing has been tested in past studies for numerous relationships but for particular research model as used in current study was never tested before, with the support of AET. Therefore, testing the mediating role of PW among the relationships of both predictors and a criterion of current research is sound contribution for knowledge in theoretical and contextual regards.

Finally, to explore the moderating role of POS between both independent variables and the dependent variable, H5 and H6 were formulated. As per results it is clear that POS moderated the relationship of WB and EP, further POS also moderated the relationship between WI and EP. So, H5 and H6 both have got support from results. Role of POS among different relationships was found point of interest for different researchers in past studies, but in the case of health sector of AJ&K, Pakistan this study is first attempt to test the moderating role of POS between two relationships: WB-EP and WI-EP. Thus, testing and reporting the results of H5 and H6 are also sound contribution for AET in theoretical and contextual regard.

## Implications

The current study has numerous theoretical and practical implications. As current study has taken psychological wellbeing as a mediator and perceived organizational support as a moderator and the effect of bullying and incivility on employee performance is studies through mediating moderating mechanism this theoretical model has not only ensure the nature of relationship between study variables but it will also encourage change in practice.

Framework of current paper describes a powerful model that how negative behaviors can affect the performance of organization. Current paper is also enriched with the information on employee psychological wellbeing and our paper emphasizes that presence of psychological wellbeing in the organization can also enhance the performance of workers. The conclusions of current paper can also help future researchers to use them for further validations in different study environments.

Furthermore, for organizational practice this study could be a helpful source by hypothesizing the impact of bullying and incivility on performance with the presence of mediating moderating mechanism. Perceived organizational support for workers could be a source to mitigate the effect of negative behaviors and which can increase the wellbeing of employees. The consequences of the bullying could be overcome by action and batter policy development like strict rules against negativity, zero tolerance policy, conducting workshops on ethical and moral building, creating an organizational environment which is suitable for the psychological growth of employees, and current paper could pave path for these actions.

## Supporting information

S1 Appendix Questionnaire. (DOCX)

S1 File (SAV)
