# Supplementary material for: Role of workplace bullying and workplace incivility for employee performance: Mediated-moderated mechanism
Source: PLoS One. 2024 Jan 30;19(1):e0291877. doi: 10.1371/journal.pone.0291877 (PMC10826965; doi:10.1371/journal.pone.0291877)
Supplement: S1 Appendix — (DOCX) [file pone.0291877.s001.docx]

**Appendix 1**

**Questionnaire for Nursing Staff (Female)**

**Dear Respondent,**

Thank you for agreeing to be a part of this research initiative. I am Shahid Mehmood, a student of PhD (Management Sciences). I am surveying for my thesis. You are requested to please spare your precious time and fill the questionnaire. I assure you that the information obtains from the survey will not be disclosed and will only be used for research purpose.

**Gender:**

| Male | Female |
| --- | --- |

**Age Group:**

| 20-35 | 36-45 | 46-55 | 56-60 | 61 and above |
| --- | --- | --- | --- | --- |

**Experience:**

| 1-5 | 6-10 | 11-15 | 16-20 | 20and above |
| --- | --- | --- | --- | --- |

**Qualification:**

| Inter. | Bachelors | Masters | M.Phil | PhD |
| --- | --- | --- | --- | --- |

For each item of the statement below, please indicate the extent to which you agree or disagree with the following statement by (**✔**) the appropriate number as per following rating scale where:

| **Strongly Disagree** | **Disagree** | **Neutral** | **Agree** | **Strongly Agree** |
| --- | --- | --- | --- | --- |
| 1 | 2 | 3 | 4 | 5 |

Note:- Please tick (**✔**) one option.

**Section I.**

**The following statements relate to your opinion about workplace bullying. For each item of the statements below, please indicate the extent of your agreement and disagreement by ticking (✔) the appropriate number.**

| 1 | Someone withholding information which affects your performance | 1 | 2 | 3 | 4 | 5 |
| --- | --- | --- | --- | --- | --- | --- |
| 2 | Being humiliated or ridiculed in connection with your work | 1 | 2 | 3 | 4 | 5 |
| 3 | competence of level your below work do to ordered Being | 1 | 2 | 3 | 4 | 5 |
| 4 | Having key areas of responsibility removed or replaced with more trivial or unpleasant tasks. | 1 | 2 | 3 | 4 | 5 |
| 5 | you about rumors and gossip of Spreading | 1 | 2 | 3 | 4 | 5 |
| 6 | Coventry’. to ‘sent being or excluded ignored, Being | 1 | 2 | 3 | 4 | 5 |
| 7 | Having insulting or offensive remarks made about your person (i.e. habits and background), your life private your or attitudes | 1 | 2 | 3 | 4 | 5 |
| 8 | Being shouted at or being the target of spontaneous anger (or rage) | 1 | 2 | 3 | 4 | 5 |
| 9 | Intimidating behaviour such as finger-pointing invasion of personal space, shoving, blocking/barring the way. | 1 | 2 | 3 | 4 | 5 |
| 10 | job your quit should you that others from signals or Hints | 1 | 2 | 3 | 4 | 5 |
| 11 | mistakes. or errors your of reminders Repeated | 1 | 2 | 3 | 4 | 5 |
| 12 | Being ignored or facing a hostile reaction when you approach | 1 | 2 | 3 | 4 | 5 |
| 13 | effort and work your of criticism Persistent | 1 | 2 | 3 | 4 | 5 |
| 14 | ignored views and opinions your Having | 1 | 2 | 3 | 4 | 5 |
| 15 | Practical jokes carried out by people you don’t get on with | 1 | 2 | 3 | 4 | 5 |
| 16 | Being given tasks with unreasonable or impossible targets or deadlines | 1 | 2 | 3 | 4 | 5 |
| 17 | Having allegations made against | 1 | 2 | 3 | 4 | 5 |
| 18 | work your of monitoring Excessive | 1 | 2 | 3 | 4 | 5 |
| 19 | Pressure not to claim something which by right you are entitled to (e.g. sick leave, holiday entitlement, travel expenses) | 1 | 2 | 3 | 4 | 5 |
| 20 | sarcasm and teasing excessive of subject the Being | 1 | 2 | 3 | 4 | 5 |
| 21 | workload unmanageable an to exposed Being | 1 | 2 | 3 | 4 | 5 |
| 22 | abuse actual or abuse physical or violence of Threats | 1 | 2 | 3 | 4 | 5 |
| **Section II.**  **The following statements relate to your opinion about workplace incivility. For each item of the statements below, please indicate the extent of your agreement and disagreement by ticking (✔) the appropriate number.** | | | | | | |
| 1 | ways? some in you to condescending was or down you Put | 1 | 2 | 3 | 4 | 5 |
| 2 | you? about remarks derogatory or rude, demeaning, Made | 1 | 2 | 3 | 4 | 5 |
| 3 | Addressed you in unprofessional terms, either publicly or privately? | 1 | 2 | 3 | 4 | 5 |
| 4 | Made jokes at your expense? | 1 | 2 | 3 | 4 | 5 |
| 5 | Yelled, shouted, or swore at you? | 1 | 2 | 3 | 4 | 5 |
| 6 | Paid little attention to a statement you made or showed little interest in your opinion? | 1 | 2 | 3 | 4 | 5 |
| 7 | Ignored or excluded you from professional camaraderie? | 1 | 2 | 3 | 4 | 5 |
| 8 | Doubted your judgment in a matter over which you have responsibility? | 1 | 2 | 3 | 4 | 5 |
| 9 | you? to speak to failed or you Ignored | 1 | 2 | 3 | 4 | 5 |
| **Section IV.**  **The following statements relate to your opinion about perceived organizational support. For each item of the statements below, please indicate the extent of your agreement and disagreement by ticking (✔) the appropriate number.** | | | | | | |
| 1 | well-being. it’s to contribution my values organization The | 1 | 2 | 3 | 4 | 5 |
| 2 | If the organization could hire someone to replacement a lower salary it would do so. | 1 | 2 | 3 | 4 | 5 |
| 3 | me. from effort extra any appreciate to fails organization The | 1 | 2 | 3 | 4 | 5 |
| 4 | values. and goals my considers strongly organization The | 1 | 2 | 3 | 4 | 5 |
| 5 | The organization would understand a long absence due to my illness. | 1 | 2 | 3 | 4 | 5 |
| 6 | me from complaint any ignore would organization The | 1 | 2 | 3 | 4 | 5 |
| 7 | The organization disregards my best interests when it makes decisions that affect me. | 1 | 2 | 3 | 4 | 5 |
| 8 | Help is available from the organization when I have a problem. | 1 | 2 | 3 | 4 | 5 |
| 9 | well-being. my about cares really organization The | 1 | 2 | 3 | 4 | 5 |
| 10 | The organization is willing to extend itself to help me perform my job to the best of my ability. | 1 | 2 | 3 | 4 | 5 |
| 11 | The organization would fail to understand my absence due to a personal problem. | 1 | 2 | 3 | 4 | 5 |
| 12 | If the organization found a more efficient way to get my job done, they would replace  me. | 1 | 2 | 3 | 4 | 5 |
| 13 | part. my on mistake honest an forgive would organization The | 1 | 2 | 3 | 4 | 5 |
| 14 | It would take only a small decrease in my performance for the organization to want to  me. replace | 1 | 2 | 3 | 4 | 5 |
| 15 | The organization feels there is little to be gained by employing me for the rest of my  career. | 1 | 2 | 3 | 4 | 5 |
| 16 | The organization provides me little opportunity to move up the ranks. | 1 | 2 | 3 | 4 | 5 |
| 17 | Even if I did the best job possible, the organization would fail to notice. | 1 | 2 | 3 | 4 | 5 |
| 18 | The organization would grant a reasonable request for a change in my working  conditions. | 1 | 2 | 3 | 4 | 5 |
| 19 | If I were laid off, the organization would prefer to hire someone new rather than take me  back. | 1 | 2 | 3 | 4 | 5 |
| 20 | The organization is willing to help me when I need a special favor. | 1 | 2 | 3 | 4 | 5 |
| 21 | The organization cares about my general satisfaction at work. | 1 | 2 | 3 | 4 | 5 |
| 22 | If given the opportunity, the organization would take advantage of me. | 1 | 2 | 3 | 4 | 5 |
| 23 | The organization shows very little concern for me. | 1 | 2 | 3 | 4 | 5 |
| 24 | If I decided to quit, the organization would try to persuade me to stay. | 1 | 2 | 3 | 4 | 5 |
| 25 | The organization cares about my opinions. | 1 | 2 | 3 | 4 | 5 |
| 26 | The organization feels that hiring me was a definite mistake. | 1 | 2 | 3 | 4 | 5 |
| 27 | work. at accomplishments my in pride takes organization The | 1 | 2 | 3 | 4 | 5 |
| 28 | me. about than profit a making about more cares organization The | 1 | 2 | 3 | 4 | 5 |
| 29 | The organization would understand if I were unable to finish a task on time. | 1 | 2 | 3 | 4 | 5 |
| 30 | If the organization earned a greater profit, it would consider increasing my salary. | 1 | 2 | 3 | 4 | 5 |
| 31 | The organization feels that anyone could perform my job as well as I do. | 1 | 2 | 3 | 4 | 5 |
| 32 | The organization is concerned about paying me what I deserve | 1 | 2 | 3 | 4 | 5 |
| 33 | The organization wishes to give me the best possible job for which I am qualified. | 1 | 2 | 3 | 4 | 5 |
| 34 | If my job were eliminated, the organization would prefer to lay me off rather than job. new a to me transfer | 1 | 2 | 3 | 4 | 5 |
| 35 | The organization tries to make my job as interesting as possible. | 1 | 2 | 3 | 4 | 5 |
| 36 | My supervisors are proud that I am a part of this organization. | 1 | 2 | 3 | 4 | 5 |
| **Section V.**  **The following statements relate to your opinion about psychological well-being. For each item of the statements below, please indicate the extent of your agreement and disagreement by ticking (✔) the appropriate number.** | | | | | | |
| 1 | Have you recently been able to concentrate on whatever you’re doing? | 1 | 2 | 3 | 4 | 5 |
| 2 | worry? over sleep much lost recently you Have | 1 | 2 | 3 | 4 | 5 |
| 3 | Have you recently felt that you were playing a useful part in things? | 1 | 2 | 3 | 4 | 5 |
| 4 | Have you recently felt capable of making decisions about things? | 1 | 2 | 3 | 4 | 5 |
| 5 | strain? under constantly felt recently you Have | 1 | 2 | 3 | 4 | 5 |
| 6 | Have you recently felt you couldn’t overcome your difficulties? | 1 | 2 | 3 | 4 | 5 |
| 7 | Have you recently been able to enjoy your normal day-to-day activities? | 1 | 2 | 3 | 4 | 5 |
| 8 | problems? to up face to able been recently you Have | 1 | 2 | 3 | 4 | 5 |
| 9 | depressed? or unhappy feeling been recently you Have | 1 | 2 | 3 | 4 | 5 |
| 10 | yourself? in confidence losing been recently you Have | 1 | 2 | 3 | 4 | 5 |
| 11 | Have you recently been thinking of yourself as a worthless person? | 1 | 2 | 3 | 4 | 5 |
| 12 | Have you recently been feeling reasonably happy? | 1 | 2 | 3 | 4 | 5 |

*****Thanks for your participation*****

**Questionnaire for Supervisors**

**Dear Respondent,**

Thank you for agreeing to be a part of this research initiative. I am Shahid Mehmood, a student of PhD (Management Sciences). I am surveying for my thesis. You are requested to please spare your precious time and fill the questionnaire. I assure you that the information obtains from the survey will not be disclosed and will only be used for research purpose.

**Gender:**

| Male | Female |
| --- | --- |

**Age Group:**

| 20-35 | 36-45 | 46-55 | 56-60 | 61 and above |
| --- | --- | --- | --- | --- |

**Experience:**

| 1-5 | 6-10 | 11-15 | 16-20 | 20and above |
| --- | --- | --- | --- | --- |

**Qualification:**

| Inter. | Bachelors | Masters | M.Phil | PhD |
| --- | --- | --- | --- | --- |

For each item of the statement below, please indicate the extent to which you agree or disagree with the following statement by (**✔**) the appropriate number as per following rating scale where:

| **Strongly Disagree** | **Disagree** | **Neutral** | **Agree** | **Strongly Agree** |
| --- | --- | --- | --- | --- |
| 1 | 2 | 3 | 4 | 5 |

Note:- Please tick (**✔**) one option.

**Section I**

**The following statements relate to your opinion about employee performance. For each item of the statements below, please indicate the extent of your agreement and disagreement by ticking (✔) the appropriate number.**

| 1 | How often this employee does adequately completes assigned duties? | 1 | 2 | 3 | 4 | 5 |
| --- | --- | --- | --- | --- | --- | --- |
| 2 | How often this employee does fulfill responsibilities specified in job description? | 1 | 2 | 3 | 4 | 5 |
| 3 | How often this employee does perform tasks that are expected of him/her? | 1 | 2 | 3 | 4 | 5 |
| 4 | How often this employee does meets formal performance requirements of the job? | 1 | 2 | 3 | 4 | 5 |
| 5 | How often this employee does engage in activities that will directly affect his/her performance evaluation? | 1 | 2 | 3 | 4 | 5 |
| 6 | How often does this employee neglect aspects of the job he/she is obligated to perform? | 1 | 2 | 3 | 4 | 5 |
| 7 | How often this employee does successfully performs essential duties? | 1 | 2 | 3 | 4 | 5 |
| 8 | How often this employee does help others who have been absent? | 1 | 2 | 3 | 4 | 5 |
| 9 | How often this employee does help others who have heavy workloads? | 1 | 2 | 3 | 4 | 5 |
| 10 | How often this employee does assist supervisor with his/her work (when not asked)? | 1 | 2 | 3 | 4 | 5 |
| 11 | How often does this employee takes time to listen to co-workers' problems and worries? | 1 | 2 | 3 | 4 | 5 |
| 12 | How often this employee does goes out of way to help new employees? | 1 | 2 | 3 | 4 | 5 |
| 13 | How often this employee does takes a personal interest in other employees? | 1 | 2 | 3 | 4 | 5 |
| 14 | How often this employee does passes along information to co-workers? | 1 | 2 | 3 | 4 | 5 |
| 15 | How often this employee’s attendance at work does is above the normal. | 1 | 2 | 3 | 4 | 5 |
| 16 | How often this employee does give advance notice when unable to come to work? | 1 | 2 | 3 | 4 | 5 |
| 17 | This employee does not take undeserved work breaks? | 1 | 2 | 3 | 4 | 5 |
| 18 | How often this employee does spend great deal of time with personal phone conversations? | 1 | 2 | 3 | 4 | 5 |
| 19 | How often does this employee complain about insignificant things at work? | 1 | 2 | 3 | 4 | 5 |
| 20 | How often this employee does conserve and protects organizational property? | 1 | 2 | 3 | 4 | 5 |
| 21 | How often this employee does adhere to informal rules devised to maintain order? | 1 | 2 | 3 | 4 | 5 |

*****Thanks for your participation*****
